# Charged Lipids Influence Phase Separation in Cell-Sized Liposomes Containing Cholesterol or Ergosterol

**DOI:** 10.3390/membranes12111121

**Published:** 2022-11-09

**Authors:** Tsuyoshi Yoda

**Affiliations:** 1 Hachinohe Industrial Research Institute, Aomori Prefectural Industrial Technology Research Center, 1-4-43 Kita-inter-kogyodanchi, Hachinohe City 039-2245, Aomori, Japan; tsuyoshi_yoda@aomori-itc.or.jp; Tel.: +81-178-21-2100; 2 The United Graduate School of Agricultural Sciences, Iwate University, 3-18-8 Ueda, Morioka City 020-8550, Iwate, Japan

**Keywords:** ergosterol, cholesterol, signal transduction, liposomes, charged lipids

## Abstract

Positively charged ion species and charged lipids play specific roles in biochemical processes, especially those involving cell membranes. The cell membrane and phase separation domains are attractive research targets to study signal transduction. The phase separation structure and functions of cell-sized liposomes containing charged lipids and cholesterol have been investigated earlier, and the domain structure has also been studied in a membrane model, containing the yeast sterol ergosterol. The present study investigates phase-separated domain structure alterations in membranes containing charged lipids when cholesterol is substituted with ergosterol. This study finds that ergosterol increases the homogeneity of membranes containing charged lipids. Cholesterol-containing membranes are more sensitive to a charged state, and ergosterol-containing liposomes show lower responses to charged lipids. These findings may improve our understanding of the differences in both yeast and mammalian cells, as well as the interactions of proteins with lipids during signal transduction.

## 1. Introduction

Minerals are one of the most important nutrient groups. Previously, the authors extracted mineral elements from cultivated oysters and measured their concentrations [1,2]. At that time, calcium, sodium, zinc, magnesium, and potassium were measured. Minerals such as calcium and sodium exist as ions in cells. Calcium ions are known as signal transduction messengers and display increased levels when the signal is “on”, and reduced levels when the signal is “off”. Mineral species often exist as positive ions under physiological conditions. Each positive ion species, such as sodium or potassium, has specific roles to play in order to activate motor proteins in microorganisms [3]. The cell membranes are composed of a phospholipid bilayer [4], containing various proteins, together with unsaturated and saturated phospholipids and sterols. The cell membrane not only separates the outside from the inside of the cells, but is also involved in the transmission of signals. The raft model [5,6] has been proposed to explain the emergence of a domain structure from the phase separation of membrane lipids. The sterols present in the membrane bilayer are mainly cholesterol and ergosterol in animal and fungal cells, respectively.

It has been reported that calcium ion concentration-dependent signal transduction and the assembly and dispersion of a domain rich in cholesterol and saturated lipids, i.e., the raft domain [5,6], exists in the cell membrane and is linked to the switching of signal transduction [7]. Cell membranes contain charged lipids [8] and the phase separation structures and functions of cell membranes containing charged lipids have been studied using cell-sized liposomes [9,10]. The domain structure has also been observed in a model membrane containing ergosterol from yeast cells, suggesting that the raft structure could be reproduced [11]. Conversely, in experiments using actual yeast cells, fluorescence localization was observed in membranes of the cells labeled with the sterol-binding fluorescent reagent filipin, suggesting the presence of ergosterol in membranes [12]. It has been reported that membrane proteins display a conformational change when liposomes contain ergosterol instead of cholesterol [13]. Therefore, the mechanism underlying membrane changes during sterol presence has been investigated. It has been shown earlier that the liquid-ordered (Lo) phase is easily formed in presence of ergosterol in nanoscale liposomes [14,15,16]. Furthermore, ergosterol reportedly alters liposome morphology in cell-sized liposomes [17] and affects domain structure with temperature fluctuations [18,19].

Herein, we investigate the effects on the phase-separated domain structure in membranes containing charged lipids when cholesterol is substituted with ergosterol. This study provides important information on differences in cellular processes, such as protein function and regulation, during protein–lipid interactions in membranes.

## 2. Materials and Methods

### 2.1. Materials

**Figure 1:**
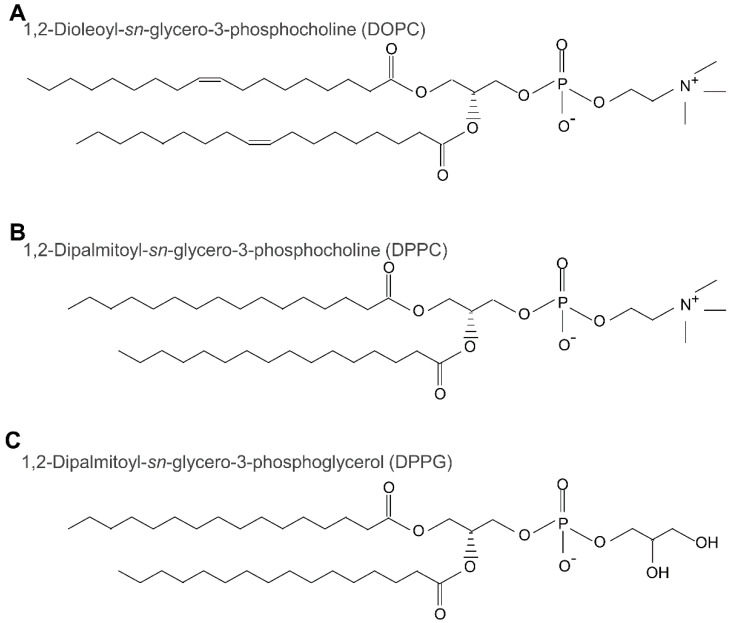
The structures of three lipids: 1,2-dioleoyl-*sn*-glycero-3-phosphocholine (DOPC, **A**); 1,2dipalmitoyl-*sn*-glycero-3-phosphocholine (DPPC, **B**); and 1,2-dipalmitoyl-*sn*-glycero-3-phosphoglycerol (DPPG, **C**).

**Figure 2:**
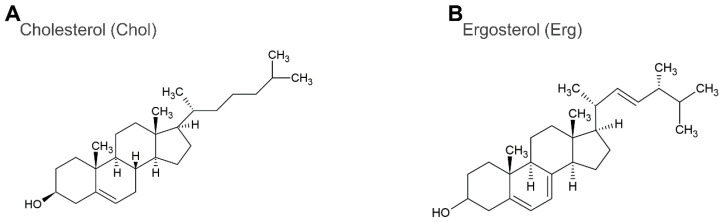
The structures of cholesterol (Chol, **A**), and ergosterol (Erg, **B**).

All lipids (Figure 1) and sterols (Figure 2) used in this study are shown respectively. 1,2-Dioleoyl-*sn*-glycero-3-phosphocholine (DOPC), 1,2-dipalmitoyl-*sn*-glycero-3-phosphocholine (DPPC), cholesterol (Chol), and ergosterol (Erg) were purchased from Tokyo Chemical Industry Co., Ltd. (Tokyo, Japan). Further, 1,2-dipalmitoyl-*sn*-glycero-3-phosphoglycerol (DPPG) was purchased from Cayman Chemical Company (Ann Arbor, MI, USA). The red fluorophore-labeled lipid, Lissamine™ Rhodamine B 1,2-dihexadecanoyl-*sn*-glycero-3-phosphoethanolamine, triethylammonium salt (Rhodamine DHPE) was obtained from Invitrogen (Carlsbad, CA, USA). The blue fluorescence-labeled dye for sterol detection, BODIPY-cholesterol, was purchased from Cayman Chemical Company. Ultrapure water obtained from the RFD240NC purification system (ADVANTEC, Tokyo, Japan) was used for reagent preparation and glassware cleaning. Acetone was purchased from Wako Pure Chemical (Osaka, Japan). Chloroform was purchased from Kanto-chemical (Tokyo, Japan). Sodium chloride (NaCl) was purchased from Fujifilm Wako Pure Chemical (Osaka, Japan). Finally, pH test paper 073200 was purchased from Whatman (Whatman, Maidstone, UK).

### 2.2. Liposome Preparation Protocol

Several different types of liposomes were prepared, including giant unilamellar vesicles (GUV) and model membranes/liposomes. A slightly modified version of the method of natural swelling from dry lipid films was used as outlined in previous studies [9,20,21,22,23,24,25,26,27,28,29,30,31,32,33,34]. Since unilamellar vesicle formation is highly sensitive to the preparation conditions, samples were prepared under well-controlled physical conditions, as described below. Glass test tubes were thoroughly washed with acetone and air-dried. Further, mixtures of lipids and Rhodamine DHPE were dissolved in chloroform, in these glass test tubes under argon gas. The tubes were then dried under vacuum for 3 h to form thin lipid films. Next, some of the films were hydrated overnight with ultrapure water at room temperature (20 ± 2.0 °C) to reach the final concentration of 0.2 mM total lipid, 0.1 μM Rhodamine DHPE, and 0.2 μM BODIPY-cholesterol in the hydrated film. Another batch of films was hydrated with ultrapure water or 10 mM NaCl solution at 50 °C for over 3 h using a drying oven (DV 41, Yamato Scientific Co., Ltd., Tokyo, Japan) for GUV formation. The lipids should appear in a fluid state where swelling occurs at T > Tm (melting temperature) of DPPC at 41 °C [35,36]. During hydration, the test tubes were double wrapped with parafilm and aluminum foil to prevent oxidation and to preserve fluorescence. After hydration, the test tubes were stored in a drawer at a constant room temperature in the dark until the time of observation, which would take place within a week.

### 2.3. Microscopic Observation

A 6 μL sample of each liposome solution, prepared as described above, was placed in a silicon well (0.2 mm depth) on a glass slide, and covered with a small cover slip. All solutions of liposomes had pH values around 7.0. Domain liposomes were then observed using a fluorescence microscope (BX51; Olympus, Tokyo, Japan) at room temperature, which was maintained using a thermal plate (TPi-X; Tokai-Hit, Fujinomiya, Japan). Images of phase-separated liposomes were obtained using a microscope-attached digital camera (WRAYCAM-VEX830; Wraymer, Osaka, Japan). At least 60 liposomes were observed for each type of lipid mixture. Samples were chosen randomly from cell-sized liposomes (~10 μm diameter). Observations made on the prepared liposomes, at least three different times, confirmed that the preparation was highly reproduceable.

### 2.4. Statistical Analysis

The data of the phase separation ratios of liposomes are expressed in terms of the mean and standard error; the data were analyzed using Microsoft Excel. One-way ANOVA in Excel was used to assess the differences, for comparison of the phase separation ratio of liposomes in each condition.

## 3. Results and Discussion

**Figure 3:**
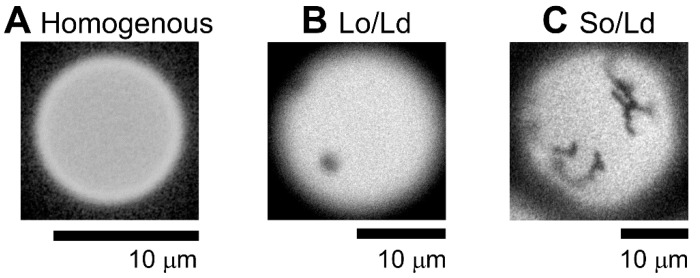
Representative microscopic images of multicomponent liposomes made with cholesterol as the sterol component, featuring three different phase-separated lipid domains. (**A**) Homogenous (DPPC:DOPC:Chol, 40:40:20), (**B**) Lo/Ld (DPPC:DOPC:Chol, 40:40:20), and (**C**) So/Ld (DPPG:DPPC:DOPC:Chol, 15:25:40:20). All experiments were performed at 20.0 °C. The images were recorded by the author. Abbreviations: DPPC, 1,2-dipalmitoyl-*sn*-glycero-3-phosphocholine; DOPC, 1,2-dioleoyl-*sn*-glycero-3-phosphocholine; DPPG, 1,2-dipalmitoyl-*sn*-glycero-3-phosphoglycerol; Chol, cholesterol; Lo/Ld, liquid-ordered/liquid-disordered; So/Ld, solid-ordered/liquid-disordered.

There are three kinds of phase-separated liposomes. First, the homogeneous phase (Figure 3A), second, liquid-ordered (Lo)/liquid-disordered (Ld) phase separation (Figure 3B), and third, solid-ordered (So) and Ld phase separation (Figure 3C). The lipid compositions of both, homogenous and Lo/Ld was DPPC:DOPC:Chol, 40:40:20; and that of So/Ld was DPPG:DPPC:DOPC:Chol, 25:15:40:20, respectively. According to previous studies, phase separation is influenced the mixing fraction of lipids [37]. A wide variation in the mixed fraction of lipids compartment during the liposome preparation has been reported earlier [38]. Therefore, the same condition (DPPC:DOPC:Chol, 40:40:20) produce two different phase-separated domains (Figure 3). These liposomes were observed in previous studies [9]. Previous reports have indicated that three-phase coexistence can be produced [9,18,37,39]. Sample observation identified more than 60 vesicles in each condition, and results are shown as an average of three observations with error bars as standard errors. All experiments were performed in triplicates to confirm replication of the results. Based on the phase diagram for DOPC/DPPC/Chol, it is near the region in which all three phases coexist in a single liposome [40,41]. Some studies have reported that the three phases co-exist in a single liposome made by charged lipids [9,42]. Since this observation may present limitations for the staining of a single phase using Rhodamine DHPE, we attempted to observe three co-existing phases by staining with BODIPY-cholesterol dye; however, microscopic conditions in our institute could not detect three coexisting phases within a single liposome, since liposome positions varied with changing irradiation wavelength. This was caused during changing of the filter, which in turn caused some vibrations that changed the position of liposome. This can be avoided by using confocal microscope, or liposome can be fixed using coating material, which we plan to do in future. This study considered only the homogenous, Lo/Ld, and Ld/So domains.

**Figure 4:**
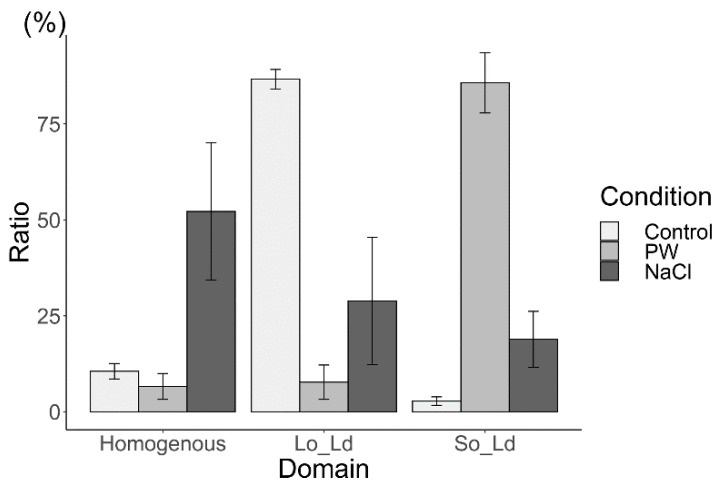
The percentages of phase-separated structures (homogenous, Lo/Ld, and So/Ld in DPPC:DOPC:Chol (40:40:20), Control, bright grey); DPPG:DPPC:DOPC:Chol (15:25:40:20), made in pure water (PW, grey)); and DPPG:DPPC:DOPC:Chol (15:25:40:20) made in a 10 mM sodium chloride solution (NaCl, dark grey), respectively, performed at 20.0 °C. Condition PW means the number ratio of homogeneous, Lo/Ld, So/Ld liposomes observed in pure water, and condition NaCl means those observed in a 10 mM NaCl solution. Each bar represents the average value of three samples; standard errors are shown as error bars. Abbreviations: DPPC, 1,2-dipalmitoyl-*sn*-glycero-3-phosphocholine; DOPC, 1,2-dioleoyl-*sn*-glycero-3-phosphocholine; DPPG, 1,2-dipalmitoyl-*sn*-glycero-3-phosphoglycerol; Chol, cholesterol; Lo/Ld, liquid-ordered/liquid-disordered; So/Ld, solid-ordered/liquid-disordered.

The effects of charged lipids on Lo/Ld membranes containing cholesterol and ergosterol were initially investigated under cholesterol conditions. As in a previous report by Himeno et al. [9], the DOPC/DPPC/Chol (40/40/20) liposome was used as a control for the Lo/Ld phase separation (Figure 4, bright grey). In the present study, the ratios for homogenous, Lo/Ld, and So/Ld were, 10.6%, 86.7%, and 2.7%, respectively; these results are in agreement with a previous study [37]. To investigate the interaction of charged lipids, DPPG was used instead of DPPC (DPPG:DPPC:DOPC:Chol, 15:25:40:20), which increased the formation of So/Ld domains (Figure 4, grey). The ratio of Lo/Ld decreased (Figure 4). Thereafter, the charge was screened using NaCl. Liposomes made from DPPG:DPPC:DOPC:Chol (15:25:40:20) in a NaCl solution were observed. The ratio of the homogenous phase increased (Figure 4, dark grey), that of the Lo/Ld phase increased, and that of the So/Ld phase decreased. The differences in the ratio of phase-separated liposomes containing charged lipids were statistically significant in each condition (one-way ANOVA, *p* < 0.05). Such trends show that the neutral lipid state is favored after screening with NaCl. These phenomena are consistent with previous results [9].

**Figure 5:**
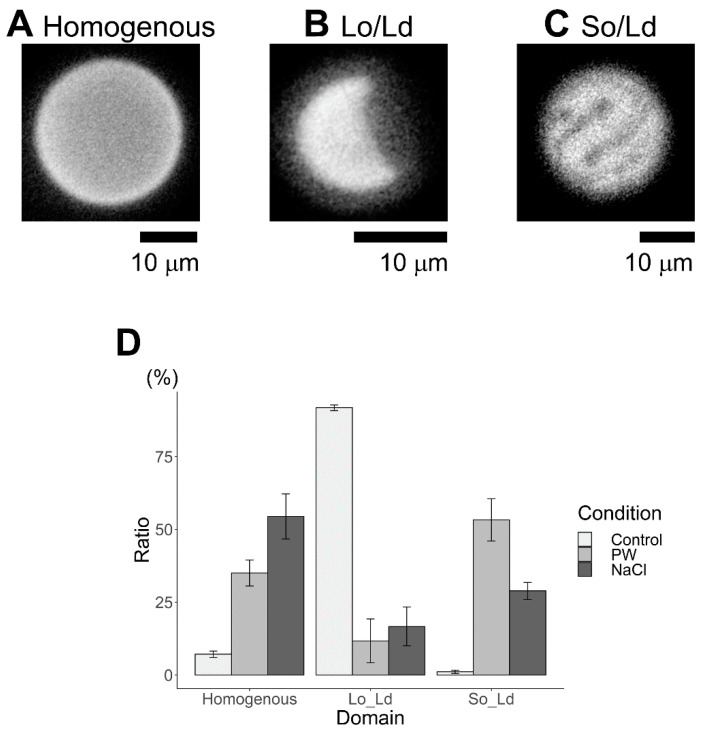
Representative microscopic images of multicomponent liposomes made with ergosterol as the sterol component, featuring three different phase-separated lipid domains (**A**,**B**), and percentages of phase-separated structures (**D**). (**A**) Homogenous (DPPG:DPPC:DOPC:Erg), 15:25:40:20 made in pure water (PW); (**B**) Lo/Ld (DPPC:DOPC:Erg), 40:40:20 made in pure water (Control), and (**C**) So/Ld (DPPG:DPPC:DOPC:Chol), 15:25:40:20 made in a sodium chloride (NaCl) solution at 20 °C. These images were taken by the author. (**D**) The percentages of phase-separated structures (homogenous, Lo/Ld, and So/Ld) in DPPC:DOPC:Erg; 40:40:20, (Control, bright grey); DPPG:DPPC:DOPC:Erg (15:25:40:20) made in pure water (PW, grey), and DPPG:DPPC:DOPC:Erg (15:25:40:20) made in a NaCl solution (NaCl, dark grey), respectively, at 20.0 °C. Condition PW means the number ratio of homogeneous, Lo/Ld, So/Ld liposomes observed in pure water and condition NaCl means those of observed in a 10 mM of NaCl solution. Each bar represents the average value of three samples; standard errors are shown as error bars. Abbreviations: DPPC, 1,2-dipalmitoyl-*sn*-glycero-3-phosphocholine; DOPC, 1,2-dioleoyl-*sn*-glycero-3-phosphocholine; DPPG, 1,2-dipalmitoyl-*sn*-glycero-3-phosphoglycerol; Chol, cholesterol; Lo/Ld, liquid-ordered/liquid-disordered; So/Ld, solid-ordered/liquid-disordered.

Next, the effects of charged lipids on Lo/Ld membranes containing ergosterol were investigated. The DOPC:DPPC:Erg (40/40/20) liposome was used as a control for Lo/Ld phase separation (Figure 5, bright grey). Using DOPC:DPPC:Chol (40:40:20) liposomes, Lo/Ld phase separation could be observed. Our finding agrees with previous results, indicating that Erg-containing cell-sized liposomes have Lo/Ld membranes, despite the slightly different lipid composition [14,15,16]. The finding is consistent with the nano-scale results of Bui et al. [14,15,16], who had reported that the Lo phase could be determined in 100 nm sized liposomes containing DOPC:DPPC:Erg (45:45:10) and DOPC:DPPC:Erg (35:35:30). Next, charged lipid DPPG was applied, instead of DPPC, and Erg-containing liposomes were observed (Figure 5, grey). The DPPG:DPPC:DOPC:Erg (15:25:40:20) liposome increased the formation of homogenous and So/Ld domains, and the ratio of Lo/Ld decreased (Figure 5). Next, the charge was screened using NaCl, and liposomes made from DPPG:DPPC:DOPC:Erg (15:25:40:20) in a NaCl solution were observed. The ratio of the homogenous phase increased (Figure 4, dark grey), the ratio of the Lo/Ld phase did not change significantly, and the ratio of the So/Ld phase decreased. Difference in ratio of phase-separated liposomes containing ergosterol were statistically significant in each condition (one-way ANOVA, *p* < 0.05). The influence of charged lipids and, a return to the neutral state were observed, since So/Ld decreased, despite the liposome change.

Comparing the charged lipid-dependent phase separation domain effects of the two sterols, the most important outcome is that Erg increases homogeneity in the charged lipid condition. The phenomenon of Erg increasing the homogeneity in the charged lipid indicates that Erg tends to support the mixing of each lipid component of the membrane. An increase in the homogeneous state would indicate that it is more energetically favorable than the phase-separated state. Although, in this case, we used negatively charged saturated lipids, unsaturated lipids, and Erg, and similar phenomena of an increase in homogenous liposomes compared to neutral lipid systems was reported earlier with negatively charged unsaturated lipids, saturated lipids, and Chol [10]. This study [10] indicated that phase separation was low for the charged membrane, due to the large energy loss caused by the high concentration of negatively charged lipids, even when the temperature was below the phase separation temperature. We assume that same molecular interaction is responsible for making Erg increase the homogeneity in the charged lipid condition in the present study. Recently, we have reported a similar result for phase separated membranes in cell-sized liposomes made from neutral lipids containing Erg, in which it was observed that Erg tended to increase the homogeneity, more so than when cholesterol was used as the sterol component [43]. Homogenous liposomes were increased after screening charged lipids using the NaCl solution. So/Ld, and Lo/Ld liposomes were reduced in liposomes containing charged lipids compared to liposomes without charged lipids in both, Chol- and Erg-containing liposomes, respectively (Figure 4 and Figure 5). The ratio of the So/Ld phase in Chol-containing liposomes with charged lipids was greater than that observed in Erg-containing liposomes with charged lipids. Taken together, Chol-containing membranes are more sensitive to the corresponding charged condition in liposomes containing charged lipids. The Lo/Ld phase ratio decreased, while that of the So/Ld phase increased, and these ratios had changed significantly after screening the charge. In contrast, the ratio of Lo/Ld and So/Ld phases in Erg-containing liposomes showed a mild response to charged lipids and screening.

Himeno et al. discussed that charged proteins and charged domains may play an important role in the selective adsorption of charged biomolecules [9]. Further, the phase separation behaviors of cell-sized liposomes containing charged lipids have been studied [44,45]. Kubsch et al. [44] reported that phase separation in liposomes containing charged lipids could be observed when liposomes are exposed to a NaCl solution, either in their inner or their outer region [44]. In the present study, although the outer and inner solutions both contained NaCl, similar results were obtained. Pataraia et al. reported apoptosis-related protein cytochrome C induced membrane phase separation in liposomes containing charged lipids [45]. The finding was useful to reveal mechanism of signal transduction, such as apoptosis based on molecular interaction between protein and the charged lipids at membrane phase separation. In actual cell, phase separated raft domains exist, and their clustering state is linked to the switching on and/or off of various signal pathways [7,46]. Previous studies have reported that cholesterol’s presence has an important role on raft clustering [7,47]. The present study may contribute to such research areas, because the sterol types influenced phase separation with changing ionic condition (Figure 4 and Figure 5) in the present study. Recently, it was found that two types of channels function in the process of lipid transfer in yeast, where one is calcium-dependent while the other is not [48]. The present study has found that changes in phase separation in membranes containing Erg, a yeast sterol, occur at a lower rate than that of membranes containing Chol, a mammalian sterol. These findings suggest that when the type of sterol changes, the phase separation, and signal transduction may also change significantly. However, there are regulatory mechanisms controlling protein functions, based on the interaction between proteins and lipids in presence of ions and charges [3,49]. Charged lipids and proteins and the presence of ions is fundamental to many biological processes. This study provides important insights into the effects of various sterol structures on charged lipids. However, a possible limitation may arise, since we used NaCl at a concentration of 10 mM, which is lower than that of physiological concentrations in living cells (approximately 140 mM). From the obtained results, we believe that screening using 10 mM NaCl was sufficient for Chol-containing membranes [9,10], but this concentration may be too low for Erg-containing membranes, and further research on the subject is warranted. In the cell membrane, charged lipids are distributed asymmetrically between the inner leaflet and outer leaflet [8,50,51]. In present study, lipids were distributed on the lipid bilayer almost symmetrically because the liposomes were made via natural swelling methods [51]. Hamada et al. reported the construction of asymmetric phase-separated cell-sized liposomes [52]. As asymmetric structures in cell-sized model membranes with vesicular structures would be of profound value in studies on the biologically physicochemical functions of lipid organization structures [52,53], we plan to conduct further research with asymmetrical phase-separated membranes, containing both charged lipids and several kinds of sterols in the future.

## 4. Conclusions

In the present study, changes in phase separation induced by charged lipids in membranes containing two types of sterols have been investigated, namely, cholesterol and ergosterol. Ergosterol increased the homogeneous phase in membranes containing charged lipids when compared to those containing cholesterol. Membranes containing cholesterol are more sensitive in charged conditions, and liposomes containing ergosterol showed low responses to charged lipids and NaCl screening. These findings show that certain membranes can alter their sterol structure and may further our understanding of the differences and similarities between yeast and mammalian cells, in order to improve our knowledge regarding protein and lipid interactions during signal transduction.

## Data Availability

Research data have been provided in the manuscript.
